# Physiological role of receptor activator nuclear factor-kB (RANK) in denervation-induced muscle atrophy and dysfunction

**DOI:** 10.14800/rci.1323

**Published:** 2016-05-30

**Authors:** Sébastien S. Dufresne, Antoine Boulanger-Piette, Sabrina Bossé, Jérôme Frenette

**Affiliations:** 1 Centre Hospitalier Universitaire de Québec-Centre de Recherche du Centre Hospitalier de l’Université Laval (CHUQ-CRCHUL), Université Laval, Quebec City, Quebec, G1V 4G2, Canada; 2 Département de Réadaptation, Faculté de Médecine, Université Laval, Quebec City, Quebec, G1V 4G2, Canada

**Keywords:** RANK, skeletal muscle, calcium, SERCA

## Abstract

The bone remodeling and homeostasis are mainly controlled by the receptor-activator of nuclear factor kB (RANK), its ligand RANKL, and the soluble decoy receptor osteoprotegerin (OPG) pathway. While there is a strong association between osteoporosis and skeletal muscle dysfunction, the functional relevance of a particular biological pathway that synchronously regulates bone and skeletal muscle physiopathology remains elusive. Our recent article published in the American Journal of Physiology (Cell Physiology) showed that RANK is also expressed in fully differentiated C2C12 myotubes and skeletal muscles. We used the Cre-Lox approach to inactivate muscle RANK (RANK^mko^) and showed that RANK deletion preserves the force of denervated fast-twitch EDL muscles. However, RANK deletion had no positive impact on slow-twitch Sol muscles. In addition, denervating RANK^mko^ EDL muscles induced an increase in the total calcium concentration ([Ca_T_]), which was associated with a surprising decrease in SERCA activity. Interestingly, the levels of STIM-1, which mediates Ca^2+^ influx following the depletion of SR Ca^2+^ stores, were markedly higher in denervated RANK^mko^ EDL muscles. We speculated that extracellular Ca^2+^ influx mediated by STIM-1 may be important for the increase in [Ca_T_] and the gain of force in denervated RANK^mko^ EDL muscles. Overall, these findings showed for the first time that the RANKL/RANK interaction plays a role in denervation-induced muscle atrophy and dysfunction.

## Introduction

Bone remodeling is an essential process for maintaining bone homeostasis throughout the life of an individual. It is under the control of local and systemic factors that orchestrate osteoblast/osteoclast activation ^[1–4]^. RANK, the receptor-activator of RANKL, and the OPG triad (RANK/RANKL/OPG) play important roles in fine-tuning the activity of these two bone cell types. RANKL is mainly expressed by bone marrow mesenchymal stromal cells and osteoblasts ^[5–6]^. The binding of RANKL to RANK on osteoclast precursors trimerizes its receptor and induces osteoclast differentiation and activation, resulting in bone resorption. RANK knockout impairs osteoclastogenesis and induces osteopetrosis, while the overproduction of RANKL induces osteoporosis ^[7, 8]^. OPG, the third protagonist, is mainly produced by bone marrow mesenchymal stromal cells and osteoblasts and exerts an inhibitory effect on the osteoclastic process. OPG has a high affinity for RANKL and inhibits the RANKL/RANK interaction and subsequent bone degradation. In addition to RANKL, OPG serves as a low affinity decoy receptor for TRAIL. Pre-clinical studies have suggested that this interaction increases cell survival by blocking the apoptotic effects of the RANKL/RANK interaction ^[9, 10]^. The fact that the overexpression of OPG or an exogenous OPG treatment ^[11]^ in mice results in osteopetrosis and that OPG-null mice are osteoporotic are testimony to the physiological importance of OPG ^[11]^.

Clinical studies have clearly shown that there is an association between osteoporosis and muscle atrophy and that the worsening of these conditions happens in tandem ^[12, 13]^. While bones and skeletal muscles are closely related and are mechanically linked, the possibility of dynamic molecular cross-talk between these tissues and a common signaling pathway that can efficiently control them has been underappreciated to date. Since the RANK/RANKL/OPG pathway is the most important cytokine network involved in bone biology and diseases, we postulated that this bone pathway is also involved in muscle atrophy and dysfunction. We first showed by Western blotting and confocal microscopy that C2C12 myotubes and fully differentiated skeletal muscles express RANK on the membranes of fast and slow-twitch myofibers ^[14]^. Based on the protective effect of RANK deletion on bone mass ^[15]^, we next postulated that selective muscle RANK deletion preserves muscle mass and function and favors a fast-twitch phenotype following denervation.

## Muscle phenotype and RANK

**Figure 1:**
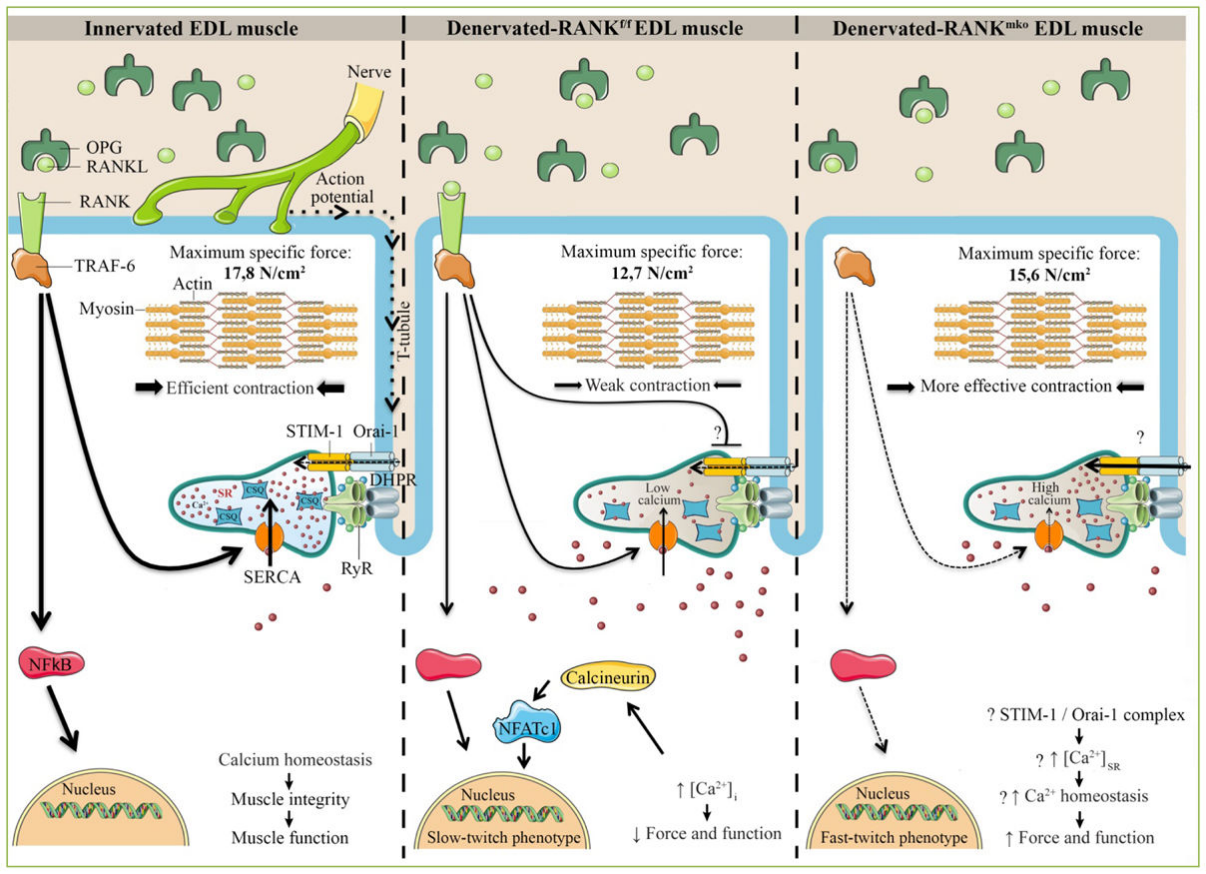
Schematic and hypothetical representation depicting the function of RANK in skeletal muscle The action potential is conducted into the interior of the muscle cell along the T-tubules where DHPR, a voltage sensor protein, triggers Ca^2+^ release from the SR through RyR and muscle contraction. Once released, SERCA pumps back the Ca^2+^ into the SR allowing muscle relaxation and preparing muscle for the next contraction. In bone cells, RANKL/RANK interaction is important for SERCA activation, [Ca^2+^]_i_ oscillations and osteoclastogenesis. In skeletal muscle, a rise in [Ca^2+^]_i_ would stimulate calcineurin, a calcium dependent protein phosphatase that subsequently dephosphorylates NFATc1 and promotes a slower muscle phenotype. Muscle specific RANK deletion (RANK^mko^) reduces SERCA activity but protects against sciatic denervation-induced muscle dysfunction and favors a fast-twitch phenotype. We speculate that STIM-1/Orai-1 complex would compensate for the lack of SERCA activity, refilling Ca^2+^ stores and improving muscle function in atrophied and denervated RANK^mko^ EDL muscles. Although the complete and precise role of muscle RANK remains poorly understood, our article in American Journal of Physiology (Cell physiology) shows that muscle RANK is an important regulator of Ca^2+^ storage, muscle phenotype, and muscle function in normal and pathological conditions.

Skeletal muscles are primarily composed of four muscle fiber types: type I fibers (slow and oxidative), type IIa fibers (fast and oxidative), and type IIx and IIb fibers (fast and glycolytic). The function largely dictates the phenotype and composition of each skeletal muscle. For example, the Sol muscle, a postural muscle, is largely composed of slow oxidative type I fibers while the EDL muscle, a phasic muscle, is almost exclusively composed of fast-twitch fibers ^[16]^. These two muscles encompass the two extremes of the phenotypic spectrum of the skeletal muscle contractile apparatus. Type I fibers play an important role in maintaining the body in an upright position, while type IIb and IIx fibers are responsive during movement and physical activity. Type IIa fibers are a hybrid of type I and type IIb fibers and can perform short or prolonged exercises. Specific exercises, immobilization, unloading, denervation, muscle diseases, or glucocorticoid treatments affect all four muscle fiber phenotypes to different degrees. For instance, decreases in mechanical loading and neuromuscular activity favor muscle atrophy and a conversion of muscle fiber phenotypes from slow to fast ^[17]^. Functional muscle overload causes a gain in muscle mass while prolonged exercise leads to the transformation of pre-existing fast-twitch muscle fibers to a slow-twitch oxidative phenotype. Type IIb fibers, the most powerful fibers, are converted to oxidative phenotype fibers (type I or IIa) and disappear through a necrotic process during myopathies, aging, and glucocorticoid treatments ^[18, 19]^. Intracellular/cytosolic Ca^2+^ ([Ca^2+^]_i_) fluctuations play a crucial role in the maintenance and adaptation of muscle phenotypes. The increase in [Ca^2+^]_i_ is caused by a Ca^2+^ influx from the extracellular space and/or Ca^2+^ release from [Ca^2+^]_i_ stores. The SR is the major releasable [Ca^2+^]_i_ store. Ca^2+^ channels and pumps maintain a steep Ca^2+^ concentration gradient between different cell compartments. Generally speaking, the spatial pattern of increased free [Ca^2+^]_i_ at rest induces the activation of calmodulin and downstream Ca^2+^ decoders, namely calcineurin and CaMKII ^[20]^. These two decoders modify gene transcription by activating transcription factors such as NFATc1 and MEF2 and transcription modulators such as HDAC and PGC-1α, promoting the slow myofiber phenotype ^[21]^ (Figure 1). Interestingly, the increase in [Ca^2+^]_SR_, is essential to activate NFATc1, while extracellular Ca^2+^ is required to maintain its activation ^[22]^ and to replenish [Ca^2+^]_i_ stores ^[23]^. Since over 97% of [Ca^2+^]_i_ is stored in the SR at rest, the measurement of total Ca^2+^ ([Ca_T_]) is an estimate of [Ca^2+^]_SR_ content ^[24]^. We used Cre-Lox technology to inactivate muscle RANK and found that the selective deletion of muscle RANK (RANK^mko^) decreases [Ca_T_] in fast-twitch EDL muscles but not in Sol muscles, when compared to RANK^flox/flox^ (RANK^f/f^) muscles. However, sciatic denervation markedly increased [Ca_T_] in RANK^mko^ EDL muscles. In addition, we discovered that denervated RANK^mko^ EDL muscles are smaller, more fatigable, and surprisingly stronger than RANK^f/f^ EDL muscles. Since force production is usually proportional to muscle size, we hypothesized that the increase in [Ca_T_], i.e., [Ca^2+^]_SR_ at rest, would favor Ca^2+^ release, increasing free [Ca^2+^]_i_ and thereby the specific force of denervated RANK^mko^ EDL muscles. The phenotypic profiles, which are consistent with these observations, showed a lower proportion of slow-twitch type I and fast-twitch type IIx fibers and a higher proportion of fast-twitch type IIa/IIb fibers in RANK^mko^ EDL muscles relative to RANK^f/f^ EDL muscles. However, muscle fatigue was significant and could not be explained by the modest changes in myofiber type in sham and denervated RANK^mko^ EDL muscles. In this context, it would be highly relevant to determine whether muscle RANK deletion influences mitochondria Ca^2+^ uptake, another important regulator of [Ca^2+^]_i_ and energy production. Our results showed for the first time that muscle RANK is an important regulator of Ca^2+^ storage, muscle phenotype, and muscle fatigue in normal and pathological conditions.

## Calcium signaling, RANK, and skeletal muscle

Muscle contraction involves the depolarization of the transverse-tubular system and activation of DHPRs, which in turn open RYR1 receptors adjacent to the SR membrane ^[25]^. This results in the rapid efflux of large amounts of Ca^2+^ into the cytoplasm and the binding of Ca^2+^ to troponin C, causing the formation of cross-bridges between actin and myosin, the shortening of the sarcomere, and force development ^[26]^. To avoid permanent contraction and allow muscle relaxation, Ca^2+^ is in part pumped back into the SR by an ATP-dependent Ca^2+^ pump called SERCA. Ca^2+^ reuptake into the SR is mainly controlled by the fast-twitch SERCA-1a and slow-twitch SERCA-2a isoforms in myofibers. The movement of Ca^2+^ is reduced when PLN is associated with SERCA. Under β-adrenergic stimulation, like the stress hormones adrenaline and noradrenaline, or sympathomimetic drugs, the phosphorylation-dependent dissociation of PLN by PKA increases SERCA activity and Ca^2+^ movement, enhancing skeletal muscle and heart functions (Figure 1). Alternatively, PLN deletion increases cardiac output by decreasing the time to peak pressure and the half-relaxation time ^[27]^, indicating that efficient Ca^2+^ mobilization and the disinhibition of SERCA are prerequisites for powerful contractions. Conversely, dysfunctional SERCA expression and activity impair [Ca^2+^]_i_ homeostasis, reducing the force production and power output of skeletal and cardiac muscles ^[28, 29]^. This is particularly true for fast-twitch muscles, like in dystrophic EDL muscles where Ca^2+^ handling is altered with the expression of the slow isoform of SERCA-2a ^[30]^. Increased Ca^2+^ influx thus activates proteolytic pathway, which in turn causes even greater Ca^2+^ influx, giving rise to a possible vicious pathological cycle in dystrophic muscles ^[31]^. Interestingly, the overexpression of SERCA mitigates muscular dystrophy and rescues cardiac function in a model of pressure overload, highlighting the importance of SERCA in muscle performance and disease ^[32, 33]^. In our paper, we report a number of intriguing and challenging observations, including the fact that RANK deletion in denervated EDL muscles increases [Ca_T_] and specific force and decreases SERCA activity. This apparent discrepancy between the increase in [Ca_T_] and the decrease in SERCA activity may be due to an alternative source of Ca^2+^ refilling the SR. Indeed, extracellular Ca^2+^ and SOCE are important for optimal muscle force production, while SOCE inhibition is associated with a decrease in contractile properties ^[34]^. More specifically, Orai-1 Ca^2+^ channels are key sensors and major contributors to Ca^2+^ entry into the SR via an interplay with STIM-1. When SR Ca^2+^ stores are depleted, STIM-1 activates Orai-1 to refill the stores ^[35]^. Additionally, Stiber *et al*. (2008) demonstrated that STIM-1^+/+^ myotubes maintain full Ca^2+^ stores whereas STIM-1^−/−^ myotubes fail to refill SR Ca^2+^ stores following chronic stimulations ^[36]^. Interestingly, we showed that denervated RANK^mko^ EDL muscles have a markedly higher STIM-1 content. Since the increase in [Ca_T_] and the decrease in SERCA activity in denervated RANK^mko^ EDL muscles are somewhat irreconcilable, we proposed that STIM-1 and Orai-1 compensate for the lack of SERCA activity, refilling SR Ca^2+^ stores in the absence of muscle RANK.

## RANKL/RANK interactions and intracellular signaling networks in bone and skeletal muscle

In bone, RANKL/RANK interactions activate TRAF-6, which then induces the activation of downstream signaling molecules and [Ca^2+^]_i_ oscillations ^[37]^. SERCA is essential for [Ca^2+^]_i_ oscillations and plays a critical role in osteoclastogenesis ^[38]^. It has recently been reported that SERCA-2a heterozygote mice (SERCA2a^+/−^) present defects in osteoclast differentiation and the suppression of RANKL-induced Ca^2+^ oscillations ^[39]^. Tmem64, a positive modulator of osteoclast differentiation, regulates RANKL-induced Ca^2+^ signaling via SERCA2a ^[40]^. In addition to SERCA, Ca^2+^ oscillations may partly derive from extracellular Ca^2+^. Gadolinium, a specific SOCE blocker, abolishes RANKL-induced Ca^2+^ oscillations ^[41]^ while the knockdown of STIM-1 significantly reduces the frequency of RANKL-induced Ca^2+^ oscillations, indicating that SOCE and RANK communicate. TRP channels are also candidates for the Ca^2+^ influx involved in RANKL-induced Ca^2+^ oscillations ^[42]^. Chamoux *et al*. (2010) showed that RANKL induces a significant increase in [Ca^2+^]_i_ from outside, presumably through the TRPV-5 Ca^2+^ channel. While an elaborate approach has been proposed to elucidate how RANKL/RANK induces Ca^2+^ oscillations in bone cells, the importance of this pathway in skeletal muscle is extremely limited. Our work reported in the American Journal of Physiology (Cell Physiology) was, to our knowledge, the first attempt to assign a regulatory role for RANKL/RANK interactions on SERCA activity in bone and muscle cells ^[14]^. The identification of downstream effectors of muscle RANK will be an important step in determining precisely how the RANKL/RANK interaction modulates SERCA activity. Bioinformatic approaches may eventually be required to improve our understanding of the cell signaling network and to determine how the RANKL/RANK interaction regulates SERCA activity and possibly other Ca^2+^ channels in skeletal muscles.

## Future directions and perspectives

We convincingly show in another publication in the American Journal of Pathology (2015) ^[43]^ that OPG-Fc (OPG fused to immunoglobulin Fc) completely restores the function of dystrophic EDL muscles and significantly improves the function of Sol and diaphragm muscles. OPG treatments also provide significant protection against eccentric-contraction-induced muscle damage. As observed with OPG-treated dystrophic mice, muscle RANK deletion preferentially protects fast-twitch fibers. It is important to note that fast-twitch fibers are essential for brief and powerful contractions. They are predominantly affected directly or indirectly in aging and in several forms of muscle disease and chronic illness, including diabetes, congestive heart failure, renal failure, chronic heart disease, and chronic obstructive pulmonary disease. Our results clearly show that the role of the RANK/RANKL/OPG pathway extends well beyond bone remodeling and osteoporosis and that skeletal muscles and other tissues may share the RANK/RANKL/OPG pathway as a common denominator.

## Abbreviations

RANK: receptor-activator of nuclear factor kB
RANKL: ligand of receptor-activator of nuclear factor kB
OPG: osteoprotegerin
TRAF-6: tumor necrosis factor associated factor-6
NFATc1: nuclear factor of activated T cells 1
MAPKs: mitogen activated protein kinases
TRAIL: tumor necrosis factor-related apoptosis-inducing ligand
Sol: soleus muscle
EDL: extensor digitorum longus muscle
[Ca^2+^]_i_: intracellular calcium
CSQ: calsequestrin
CaMKII: calmodulin-dependent protein kinase II
MEF2: myocyte enhancer factor
HDAC: histone deacetylase
PGC-1: peroxisome-proliferator-activated receptor gamma, co-activator 1
SR: sarcoplasmic reticulum
[Ca_T_]: total calcium content
RANK^mko^: specific RANK skeletal muscle deletion
RANK^f/f^: RANK^flox/flox^ skeletal muscle
DHPR: dihydropyridine receptor
RYR1: ryanodine receptor
SERCA: sarco(endo)plasmic reticulum Ca^2+^ATPase
PLN: phospholamban
PKA: protein kinase A
SOCE: store-operated Ca^2+^ entry
STIM-1: stromal interaction molecule-1
ER: endoplasmic reticulum
ECC: excitation contraction coupling
ub: ubiquitination
TRP: Transient receptor potential
Tmem64: transmembrane protein 64

## References

[1] Lacey DL, Boyle WJ, Simonet WS, Kostenuik PJ, Dougall WC, Sullivan JK (2012). Bench to bedside: elucidation of the OPG-RANK-RANKL pathway and the development of denosumab. Nat Rev Drug Discov.

[2] Atkins GJ, Findlay DM (2012). Osteocyte regulation of bone mineral: a little give and take. Osteoporos Int J Establ Result Coop Eur Found Osteoporos Natl Osteoporos Found USA.

[3] Leibbrandt A, Penninger JM (2008). RANK/RANKL: regulators of immune responses and bone physiology. Ann N Y Acad Sci.

[4] Silvestrini G, Ballanti P, Leopizzi M, Gualtieri N, Sardella D, Monnazzi P (2007). Effects of the administration of corticosterone, parathyroid hormone, or both, and of their withdrawal, on rat bone and cartilage histomorphometric parameters, and on osteoprotegerin and RANKL mRNA expression and proteins. J Mol Histol.

[5] Walsh MC, Choi Y (2014). Biology of the RANKL-RANK-OPG System in Immunity, Bone, and Beyond. Front Immunol.

[6] Baharuddin NA, Coates DE, Cullinan M, Seymour G, Duncan W (2015). Localization of RANK, RANKL and osteoprotegerin during healing of surgically created periodontal defects in sheep. J Periodontal Res.

[7] Takayanagi H (2007). The role of NFAT in osteoclast formation. Ann N Y Acad Sci.

[8] Thudium CS, Moscatelli I, Flores C, Thomsen JS, Brüel A, Gudmann NS (2014). A Comparison of Osteoclast-Rich and Osteoclast-Poor Osteopetrosis in Adult Mice Sheds Light on the Role of the Osteoclast in Coupling Bone Resorption and Bone Formation. Calcif Tissue Int.

[9] Sundaram K, Sambandam Y, Balasubramanian S, Pillai B, Voelkel-Johnson C, Ries WL (2015). STAT-6 mediates TRAIL induced RANK ligand expression in stromal/preosteoblast cells. Bone.

[10] Nelson CA, Warren JT, Wang MW-H, Teitelbaum SL, Fremont DH (2012). RANKL Employs Distinct Binding Modes to Engage RANK and the Osteoprotegerin Decoy Receptor. Structure.

[11] Bargman R, Posham R, Boskey A, Carter E, DiCarlo E, Verdelis K (2012). High- and low-dose OPG-Fc cause osteopetrosis-like changes in infant mice. Pediatr Res.

[12] Perrini S, Laviola L, Carreira MC, Cignarelli A, Natalicchio A, Giorgino F (2010). The GH/IGF1 axis and signaling pathways in the muscle and bone: mechanisms underlying age-related skeletal muscle wasting and osteoporosis. J Endocrinol.

[13] Zhu K, Yi J, Xiao Y, Lai Y, Song P, Zheng W (2015). Impaired Bone Homeostasis in Amyotrophic Lateral Sclerosis Mice with Muscle Atrophy. J Biol Chem.

[14] Dufresne SS, Dumont NA, Boulanger-Piette A, Fajardo VA, Gamu D, Kake-Guena SA (2016). Muscle RANK is a key regulator of calcium storage, SERCA activity, and function of fast-twitch skeletal muscles. Am J Physiol Cell Physiol.

[15] Xing L, Chen D, Boyce BF (2013). Mice Deficient in NF-κB p50 and p52 or RANK Have Defective Growth Plate Formation and Post-natal Dwarfism. Bone Res.

[16] Khodabukus A, Baar K (2015). Contractile and Metabolic Properties of Engineered Skeletal Muscle Derived From Slow and Fast Phenotype Mouse Muscle: In vitro mouse phenotype and function. J Cell Physiol.

[17] Däpp C, Schmutz S, Hoppeler H, Flück M (2004). Transcriptional reprogramming and ultrastructure during atrophy and recovery of mouse soleus muscle. Physiol Genomics.

[18] Arnardottir S, Borg K, Ansved T (2004). Sporadic inclusion body myositis: morphology, regeneration, and cytoskeletal structure of muscle fibres. J Neurol Neurosurg Psychiatry.

[19] Gueugneau M, Coudy-Gandilhon C, Théron L, Meunier B, Barboiron C, Combaret L (2015). Skeletal muscle lipid content and oxidative activity in relation to muscle fiber type in aging and metabolic syndrome. J Gerontol A Biol Sci Med Sci.

[20] Eilers W, Jaspers RT, de Haan A, Ferrié C, Valdivieso P, Flück M (2014). CaMKII content affects contractile, but not mitochondrial, characteristics in regenerating skeletal muscle. BMC Physiol.

[21] Norrbom J, Sundberg CJ, Ameln H, Kraus WE, Jansson E, Gustafsson T (2004). PGC-1alpha mRNA expression is influenced by metabolic perturbation in exercising human skeletal muscle. J Appl Physiol Bethesda Md 1985.

[22] Stiber JA, Tabatabaei N, Hawkins AF, Hawke T, Worley PF, Williams RS (2005). Homer modulates NFAT-dependent signaling during muscle differentiation. Dev Biol.

[23] Valdes JA, Gaggero E, Hidalgo J, Leal N, Jaimovich E, Carrasco MA (2008). NFAT activation by membrane potential follows a calcium pathway distinct from other activity-related transcription factors in skeletal muscle cells. AJP Cell Physiol.

[24] Lamboley CRH, Kake Guena SA, Touré F, Hébert C, Yaddaden L, Nadeau S (2015). New method for determining total calcium content in tissue applied to skeletal muscle with and without calsequestrin. J Gen Physiol.

[25] Delbono O (2011). Expression and regulation of excitation-contraction coupling proteins in aging skeletal muscle. Curr Aging Sci.

[26] Zhang T, Taylor J, Jiang Y, Pereyra AS, Messi ML, Wang Z-M (2015). Troponin T3 regulates nuclear localization of the calcium channel Cavβ1a subunit in skeletal muscle. Exp Cell Res.

[27] Luo W, Grupp IL, Harrer J, Ponniah S, Grupp G, Duffy JJ (1994). Targeted ablation of the phospholamban gene is associated with markedly enhanced myocardial contractility and loss of beta-agonist stimulation. Circ Res.

[28] Krishna A, Valderrábano M, Palade PT, Clark JW (2013). Rate-dependent Ca2+ signalling underlying the force-frequency response in rat ventricular myocytes: a coupled electromechanical modeling study. Theor Biol Med Model.

[29] Fajardo VA, Bombardier E, McMillan E, Tran K, Wadsworth BJ, Gamu D (2015). Phospholamban overexpression in mice causes a centronuclear myopathy-like phenotype. Dis Model Mech.

[30] Divet A, Lompré A-M, Huchet-Cadiou C (2005). Effect of cyclopiazonic acid, an inhibitor of the sarcoplasmic reticulum Ca-ATPase, on skeletal muscles from normal and mdx mice. Acta Physiol Scand.

[31] Brussee V, Tardif F, Tremblay JP (1997). Muscle fibers of mdx mice are more vulnerable to exercise than those of normal mice. Neuromuscul Disord NMD.

[32] Goonasekera SA, Lam CK, Millay DP, Sargent MA, Hajjar RJ, Kranias EG (2011). Mitigation of muscular dystrophy in mice by SERCA overexpression in skeletal muscle. J Clin Invest.

[33] Ferretti R, Marques MJ, Pertille A, Santo Neto H (2009). Sarcoplasmic-endoplasmic-reticulum Ca2+-ATPase and calsequestrin are overexpressed in spared intrinsic laryngeal muscles of dystrophin-deficient mdx mice. Muscle Nerve.

[34] Thornton AM, Zhao X, Weisleder N, Brotto LS, Bougoin S, Nosek TM (2011). Store-operated Ca(2+) entry (SOCE) contributes to normal skeletal muscle contractility in young but not in aged skeletal muscle. Aging.

[35] Kiviluoto S, Decuypere J-P, De Smedt H, Missiaen L, Parys JB, Bultynck G (2011). STIM1 as a key regulator for Ca2+ homeostasis in skeletal-muscle development and function. Skelet Muscle.

[36] Stiber J, Hawkins A, Zhang Z-S, Wang S, Burch J, Graham V (2008). STIM1 signalling controls store-operated calcium entry required for development and contractile function in skeletal muscle. Nat Cell Biol.

[37] Darnay BG, Haridas V, Ni J, Moore PA, Aggarwal BB (1998). Characterization of the intracellular domain of receptor activator of NF-kappaB (RANK). Interaction with tumor necrosis factor receptor-associated factors and activation of NF-kappab and c-Jun N-terminal kinase. J Biol Chem.

[38] Mentaverri R, Kamel S, Brazier M (2003). Involvement of capacitive calcium entry and calcium store refilling in osteoclastic survival and bone resorption process. Cell Calcium.

[39] Yang Y-M, Kim MS, Son A, Hong JH, Kim K-H, Seo JT (2009). Alteration of RANKL-Induced Osteoclastogenesis in Primary Cultured Osteoclasts From SERCA2 +/− Mice. J Bone Miner Res.

[40] Kim H, Kim T, Jeong B-C, Cho I-T, Han D, Takegahara N (2013). Tmem64 Modulates Calcium Signaling during RANKL-Mediated Osteoclast Differentiation. Cell Metab.

[41] Kim MS, Yang Y-M, Son A, Tian YS, Lee S-I, Kang SW (2010). RANKL-mediated Reactive Oxygen Species Pathway That Induces Long Lasting Ca2+ Oscillations Essential for Osteoclastogenesis. J Biol Chem.

[42] Chamoux E, Bisson M, Payet MD, Roux S (2010). TRPV-5 Mediates a Receptor Activator of NF-kappaB (RANK) Ligand-induced Increase in Cytosolic Ca2+ in Human Osteoclasts and Down-regulates Bone Resorption. J Biol Chem.

[43] Dufresne SS, Dumont NA, Bouchard P, Lavergne É, Penninger JM, Frenette J (2015). Osteoprotegerin protects against muscular dystrophy. Am J Pathol.

